# Cardiovascular Health in the Shadow of Diabetes and Metabolic Dysfunction-Associated Steatotic Liver Disease: An Emerging Paradigm

**DOI:** 10.31083/RCM43143

**Published:** 2025-11-27

**Authors:** Alfredo Caturano, Davide Nilo, Giovanni Di Lorenzo, Maria Rocco, Giuseppina Tagliaferri, Alessia Piacevole, Mariarosaria Donnarumma, Ilaria Iadicicco, Simona Maria Moretto, Carlo Acierno, Celestino Sardu, Vincenzo Russo, Marco Alfonso Perrone, Erica Vetrano, Raffaele Galiero, Raffaele Marfella, Leonilde Bonfrate, Luca Rinaldi, Caterina Conte, Ferdinando Carlo Sasso

**Affiliations:** ^1^Department of Human Sciences and Promotion of the Quality of Life, San Raffaele Roma University, 00166 Rome, Italy; ^2^Department of Advanced Medical and Surgical Sciences, University of Campania Luigi Vanvitelli, 80138 Naples, Italy; ^3^Department of Infectious Diseases, Azienda Ospedaliera Regionale San Carlo, 85100 Potenza, Italy; ^4^Sbarro Institute for Cancer Research and Molecular Medicine, Center for Biotechnology, College of Science and Technology, Temple University, Philadelphia, PA 19122, USA; ^5^Division of Cardiology, Department of Medical Translational Sciences, University of Campania Luigi Vanvitelli, 80138 Naples, Italy; ^6^Department of Cardiology and CardioLab, University of Rome Tor Vergata, 00133 Rome, Italy; ^7^Department of Medicine and Health Sciences “Vincenzo Tiberio”, Università degli Studi del Molise, 86100 Campobasso, Italy; ^8^Department of Endocrinology and Metabolic Diseases, IRCCS MultiMedica, 20099 Milan, Italy

**Keywords:** type 2 diabetes mellitus, metabolic dysfunction-associated steatotic liver disease, cardiovascular disease, insulin resistance, oxidative stress cardiometabolic risk

## Abstract

The coexistence of type 2 diabetes (T2D), metabolic dysfunction-associated steatotic liver disease (MASLD), and cardiovascular disease (CVD) defines a clinical profile that is frequently observed in clinical practice. In addition to being highly prevalent, patients with this triad of diseases experience accelerated vascular aging and poor prognosis. Insulin resistance remains the common symptom; however, the systemic impact of this extends far beyond glucose handling, shaping inflammation, oxidative stress, and endothelial dysfunction. In this review, we highlight how these intertwined conditions challenge current diagnostic frameworks and therapeutic approaches. Moreover, we discuss under-recognized aspects, such as the contribution of gut-derived metabolites and adipose dysfunction, which often remain neglected in routine care despite strong mechanistic evidence. We also summarize the potential of noninvasive tools, biomarkers, and cardioprotective agents, such as sodium–glucose cotransporter-2 (SGLT2) inhibitors, glucagon-like peptide-1 (GLP-1) receptor agonists, and tirzepatide. While promising, these agents still face gaps in translation to everyday hepatology and cardiology clinics. Our message is that prevention and care should not be compartmentalized. Instead, an integrated, patient-centered approach, with early screening and multidisciplinary management, is needed to address this complex interplay. Moreover, recognizing the shared pathways of T2D, MASLD, and CVD may help clinicians anticipate potential complications and design more effective and sustainable strategies for long-term outcomes.

## 1. Introduction

### 1.1 Type 2 Diabetes and MASLD: A High-Risk Cardiovascular Phenotype

Cardiovascular diseases (CVDs) remain the leading cause of death worldwide and their prevalence continues to rise. Between 1990 and 2019, the number of individuals living with CVD nearly doubled, and current projections anticipate a dramatic escalation in cases and deaths by 2050 [1, 2]. These trends are largely driven by the global increase in metabolic risk factors, particularly type 2 diabetes mellitus (T2D) and metabolic dysfunction-associated steatotic liver disease (MASLD) [3, 4].

T2D represents a major global health challenge, affecting hundreds of millions of people and ranking among the leading causes of premature mortality. Its systemic impact includes microvascular and macrovascular complications, chronic kidney disease, and cancer, but cardiovascular disease remains the principal cause of death in affected individuals [5, 6]. The underlying mechanisms include endothelial dysfunction, persistent inflammation, and accelerated atherosclerosis, which together markedly increase the burden of coronary artery disease, heart failure, and arrhythmias [7, 8, 9, 10]. In parallel, MASLD, formerly termed non-alcoholic fatty liver disease, has emerged as the most common chronic liver condition, affecting nearly 40% of the global adult population [11, 12]. Beyond hepatic outcomes such as cirrhosis and hepatocellular carcinoma, CVD is the leading cause of mortality in these patients [13, 14]. Shared pathophysiological features, including insulin resistance, visceral adiposity, and systemic inflammatory activity, underscore the tight interdependence between MASLD, T2D, and cardiovascular dysfunction [15, 16, 17]. The coexistence of T2D and MASLD therefore delineates a cardiovascular high-risk phenotype, where overlapping metabolic insults synergistically accelerate vascular injury, oxidative stress, and cardiac remodelling [18, 19, 20].

### 1.2 T2D and MASLD as a Cardiovascular High-Risk Phenotype

The coexistence of T2D and MASLD defines a cardiovascular high-risk phenotype because both conditions share and amplify pathophysiological mechanisms such as insulin resistance, visceral adiposity, chronic inflammation, and oxidative stress [13, 14, 20]. These synergistic processes are associated with accelerated endothelial dysfunction, atherosclerosis, and cardiac remodelling, leading to an excess burden of cardiovascular morbidity and mortality beyond the contribution of each disease alone.

### 1.3 The Reverse Impact of Cardiovascular Disease on MASLD

While MASLD and T2D substantially increase cardiovascular morbidity and mortality, growing evidence indicates that cardiovascular disease itself may also accelerate the onset and progression of MASLD. Chronic heart failure and ischemic heart disease are frequently accompanied by hepatic congestion, impaired microcirculation, and systemic inflammation, which can worsen insulin resistance and promote steatosis and fibrosis [18, 21]. Experimental and clinical data suggest that myocardial dysfunction induces neurohormonal activation and altered lipid metabolism, thereby exacerbating hepatic injury [19]. Furthermore, persistent systemic hypoperfusion and increased venous pressures in advanced CVD create a milieu that fosters hepatic inflammation and fibrogenesis [20]. This bidirectional relationship highlights the need for integrated management strategies that recognize not only the hepatic contribution to cardiovascular risk but also the detrimental effect of CVD on liver health.

## 2. Pathophysiological Mechanisms Linking Diabetes, Liver Disease, and Cardiovascular Disease

### 2.1 Insulin Resistance and Metabolic Dysfunction

MASLD is the most common chronic liver disease worldwide, and insulin resistance (IR) is widely recognized as a central driver of its pathogenesis [22]. IR promotes hepatic steatosis, systemic inflammation, and vascular injury, which together accelerate both liver damage and cardiovascular disease [23, 24]. Mechanistically, IR increases free fatty acid (FFA) flux to the liver through enhanced lipolysis and de novo lipogenesis [24, 25], while impairing mitochondrial β-oxidation. This leads to toxic lipid accumulation and excessive reactive oxygen species (ROS) formation, fuelling hepatocellular inflammation and fibrosis [24]. In parallel, reduced apolipoprotein B-100 synthesis impairs very-low-density lipoprotein assembly, further favoring triglyceride retention.

From a clinical perspective, these mechanisms may help explain why patients with diabetes and MASLD often present with vascular injury and myocardial dysfunction earlier than expected for their age. Visceral adipose tissue worsens this scenario by releasing pro-inflammatory cytokines such as tumor necrosis factor-α (TNF-α) and interleukin-6 (IL-6), along with molecules like plasminogen activator inhibitor-1 (PAI-1) and angiotensinogen, which contribute to endothelial dysfunction and vascular remodelling [26, 27]. The imbalance of adipokines adds another layer: reduced adiponectin promotes steatosis and vascular inflammation [28, 29], while elevated leptin drives hepatic fibrogenesis and vascular hypertrophy [30, 31, 32]. These maladaptive pathways illustrate how adipose dysfunction, beyond simple fat storage, becomes a mechanistic bridge linking IR, MASLD, and CVD [33, 34].

Mitochondrial dysfunction is another crucial contributor. Impaired β-oxidation and ROS accumulation sustain a state of “metaflammation” [35, 36, 37], a chronic low-grade inflammation typical of nutrient excess. Clinically, this translates into a higher incidence of endothelial dysfunction, myocardial fibrosis, and plaque instability among patients with MASLD [38, 39].

### 2.2 Chronic Inflammation and Fibrosis

Inflammation represents a second cornerstone of the MASLD-CVD connection. Pathways such as nuclear factor kappa-light-chain-enhancer of activated B cells (NF-κB) and c-Jun N-terminal kinase (JNK), activated by cytokines, toll-like receptors, and receptors for advanced glycation end-products, directly link obesity-related inflammation to both liver fibrosis and cardiovascular injury [40, 41]. FFAs amplify these signals by triggering endoplasmic reticulum (ER) stress and ROS production [14, 42, 43], perpetuating a cycle of hepatocellular damage.

In hepatocytes, ER stress activates NADPH oxidase (NOX) isoforms, stimulating hepatic stellate cells through TGF-β signalling and promoting fibrosis [44]. Kupffer cell activation, cytokine release (IL-6, TNF-α, interleukin-1 (IL-1)), and feedback loops with insulin signalling [45, 46, 47, 48] aggravate this process. On the cardiovascular side, similar inflammatory cascades foster endothelial dysfunction, macrophage infiltration, and plaque development [14, 38, 49].

Clinically, this systemic “spillover” is evident: MASLD patients frequently present with subclinical myocardial inflammation and fibrosis, even in the absence of overt coronary disease [50, 51, 52, 53]. This observation supports the concept of liver-heart cross-talk, whereby hepatic inflammation and fibrosis contribute to heart failure with preserved ejection fraction and arrhythmias.

### 2.3 Dyslipidaemia and Lipotoxicity

Atherogenic dyslipidaemia, characterized by high triglycerides, low high-density lipoprotein cholesterol (HDL-C), and small dense low-density lipoprotein (LDL), is a hallmark of T2D and MASLD [15]. The accumulation of toxic intermediates such as diacylglycerols and ceramides further impairs insulin signalling, worsening IR [54, 55]. Mitochondrial dysfunction and oxidative stress contribute to hepatocyte apoptosis and vascular injury [15, 35, 54, 55]. In practice, this translates into patients with MASLD exhibiting not only fatty liver but also heightened thrombotic risk. Elevated fibrinogen, PAI-1, and factor VII levels establish a hypercoagulable state, predisposing to acute cardiovascular events [56, 57]. Epidemiological data suggest MASLD as an independent predictor of cardiovascular morbidity and mortality, beyond traditional risk factors [58].

### 2.4 The Gut-Liver-Heart Axis

Emerging research highlights the gut microbiome as a key player in metabolic disorders including obesity, diabetes, MASLD, and CVD [59]. Gut microbial composition undergoes significant alterations in metabolic disease states. In obese and diabetic individuals, dysbiosis is characterized by reduced levels of beneficial bacteria (e.g., *Akkermansia*, *Faecalibacterium*) and an increase in pathogenic taxa [60].

#### 2.4.1 Gut Microbiota Composition in Metabolic Disease

The gut microbiota exerts its influence on distant organs through the production and release of a wide array of metabolites that enter the systemic circulation [61]. These include short-chain fatty acids (SCFAs), bile acids, ammonia, phenols, ethanol, lipopolysaccharides (LPS), and trimethylamine (TMA), all of which can modulate key physiological and pathological pathways [61, 62]. SCFAs such as acetate, propionate, and butyrate are generated via microbial fermentation of dietary fibres, primarily by *Bacteroidetes* and certain members of *Firmicutes*. These SCFAs play an essential role in maintaining gut barrier integrity and host metabolic balance by stimulating glucagon-like peptide-1 (GLP-1) secretion, improving insulin sensitivity, and exerting anti-inflammatory effects on adipose tissue and the liver [61, 62, 63].

#### 2.4.2 Bile Acids and Host Metabolism

Beyond SCFAs, bile acids constitute another major class of microbiota-derived metabolites with systemic effects. Secondary bile acids, including deoxycholic acid (DCA) and lithocholic acid (LCA), are formed in the colon through microbial transformation of primary bile acids. These bile acids act as ligands for nuclear receptors such as the farnesoid X receptor (FXR), pregnane X receptor (PXR), and G protein-coupled receptors (GPCRs). Activation of intestinal FXR, for instance, induces the expression of fibroblast growth factor 15 (FGF15 in mice, FGF19 in humans), which then signals to the liver to repress the rate-limiting enzyme cholesterol 7α-hydroxylase (CYP7A1), thereby regulating bile acid synthesis and enterohepatic circulation [64]. Disruption of this signalling can contribute to hepatic lipid accumulation and insulin resistance, key features of MASLD.

#### 2.4.3 Endogenous Ethanol and Endotoxemia

Ethanol, another important microbial metabolite, is produced through saccharolytic fermentation by certain gut bacteria, especially *Proteobacteria* and *Enterobacteriaceae*, which are found in elevated numbers in patients with obesity and non-alcoholic steatohepatitis (NASH) [65]. Endogenous ethanol production has been shown to disrupt intestinal epithelial tight junctions, increasing gut permeability and enabling the translocation of endotoxins such as LPS into the portal circulation [66]. This process activates inflammatory cascades in the liver, promoting hepatocellular injury, steatosis, and fibrosis [67]. The resulting low-grade endotoxemia contributes not only to liver disease but also to systemic inflammation, a key driver of atherosclerosis and CVD [68].

#### 2.4.4 TMAO and Cardiometabolic Risk

**Fig. 1.**
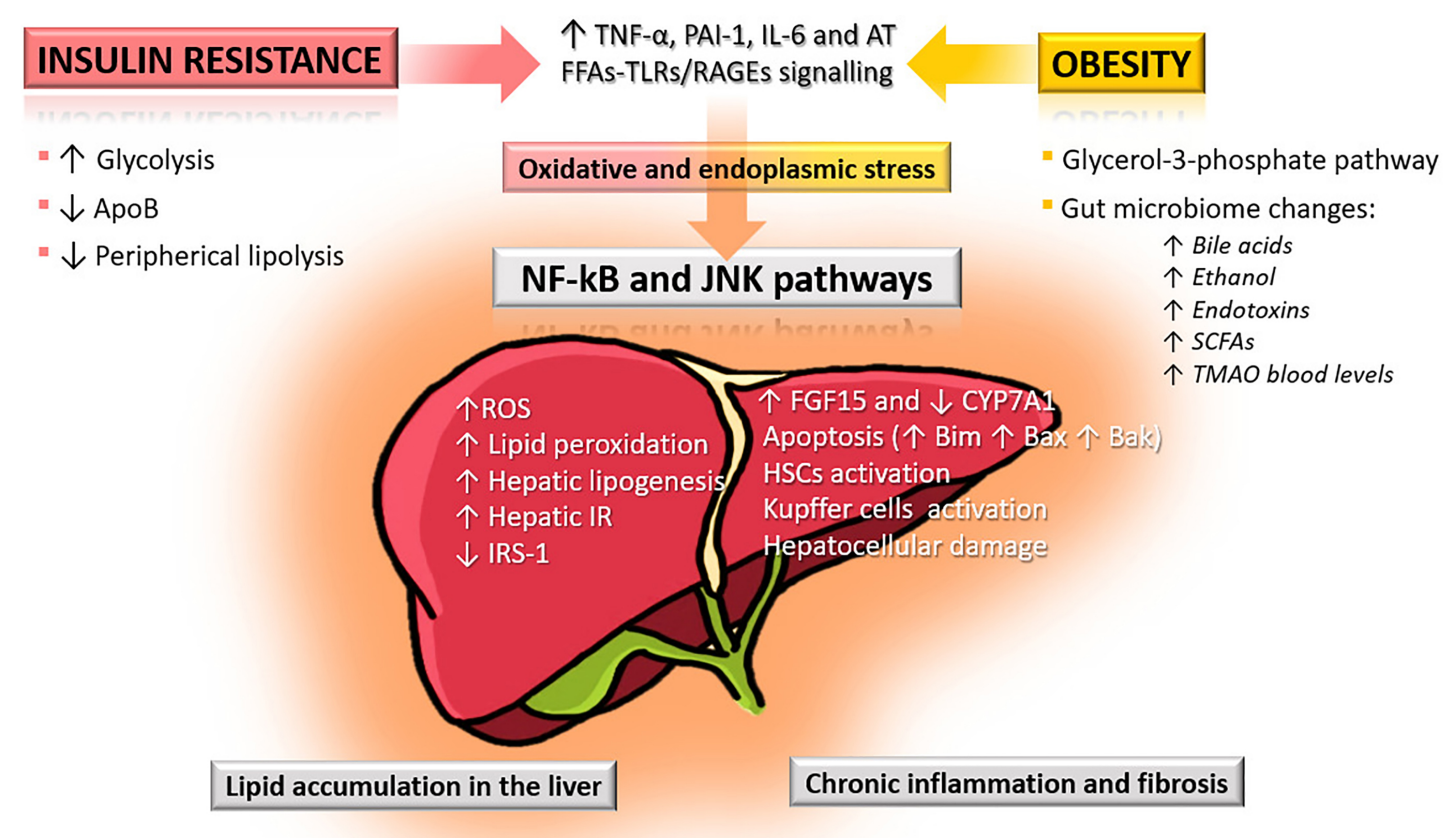
**Pathophysiological mechanisms linking insulin resistance and obesity to liver injury in MASLD**. Insulin resistance increases glycolysis and hepatic lipogenesis while reducing apolipoprotein B (ApoB) synthesis and peripheral lipolysis, leading to excess reactive oxygen species (ROS), lipid peroxidation, and hepatic insulin resistance (IR). Obesity further contributes through free fatty acid (FFA)-induced activation of toll-like receptors (TLRs) and receptors for advanced glycation end-products (RAGEs), alterations in the glycerol-3-phosphate pathway, and gut microbiome dysbiosis with elevated bile acids, ethanol, endotoxins, short-chain fatty acids (SCFAs), and trimethylamine-N-oxide (TMAO). These mechanisms converge via oxidative and endoplasmic reticulum (ER) stress, activating nuclear factor kappa-light-chain-enhancer of activated B cells (NF-κB) and c-Jun N-terminal kinase (JNK) pathways, which drive hepatocellular apoptosis, hepatic stellate cell (HSC) activation, Kupffer cell activation, reduced cholesterol 7 alpha-hydroxylase (CYP7A1), and progression from hepatic lipid accumulation to chronic inflammation and fibrosis. AT, Antithrombin; MASLD, metabolic dysfunction-associated steatotic liver disease; PAI-1, plasminogen activator inhibitor-1; FGF15, fibroblast growth factor 15; IRS-1, insulin receptor substrate 1; IL-6, Interleukin-6. ↑ increased, ↓ decreased.

**Fig. 2.**
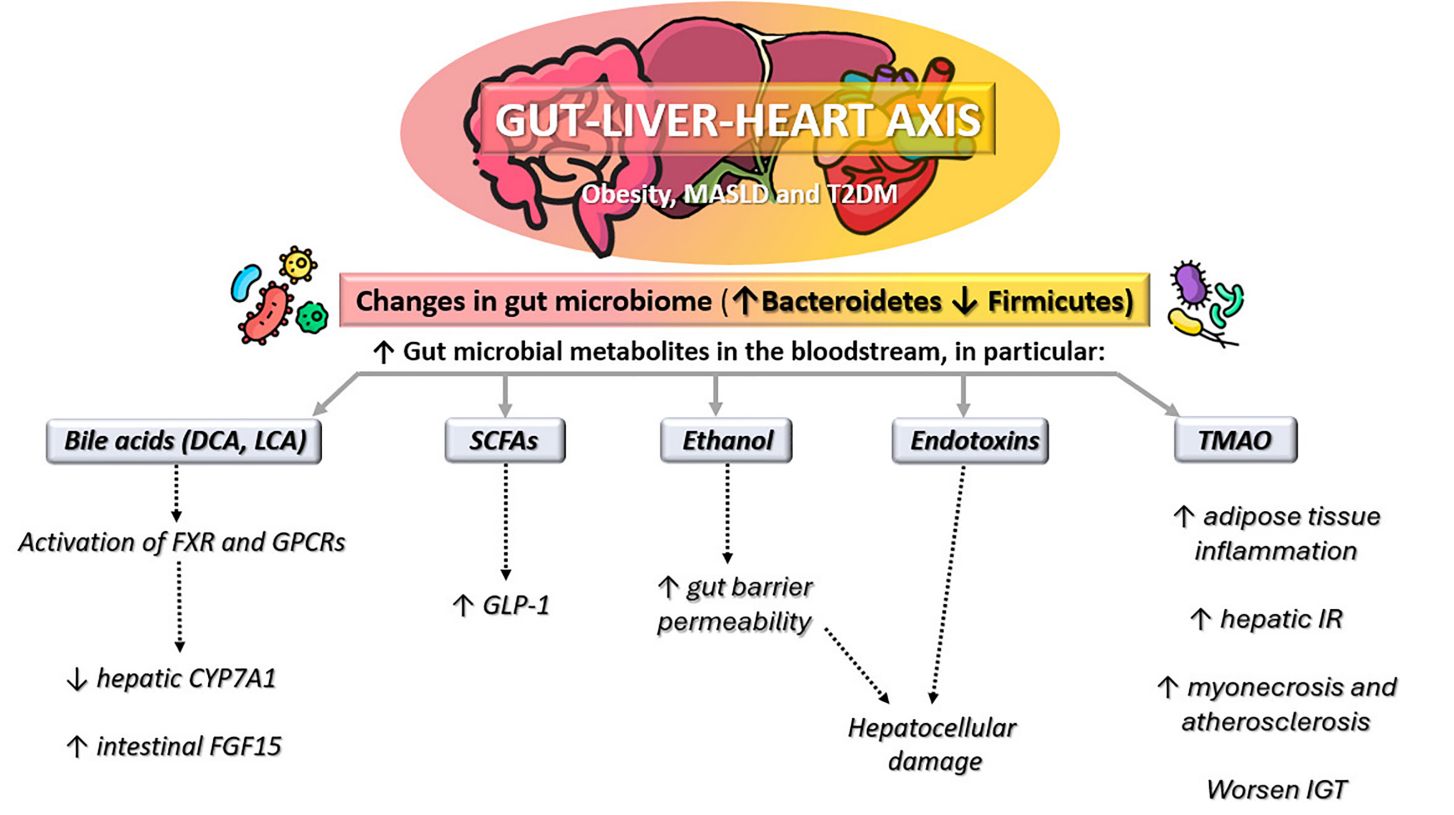
**Gut-liver-heart axis in obesity, metabolic dysfunction-associated steatotic liver disease (MASLD), and type 2 diabetes mellitus (T2DM)**. Altered gut microbiome composition (increased *Bacteroidetes*, decreased *Firmicutes*) leads to higher levels of gut microbial metabolites in the bloodstream, including bile acids (deoxycholic acid [DCA]; lithocholic acid [LCA]), short-chain fatty acids (SCFAs), ethanol, endotoxins, and trimethylamine-N-oxide (TMAO). These changes promote glucagon-like peptide-1 (GLP-1) modulation, gut barrier dysfunction, hepatocellular damage, adipose tissue inflammation, hepatic insulin resistance (IR), impaired glucose tolerance (IGT), and cardiovascular injury. FXR, farnesoid X receptor; GPCRs, G protein-coupled receptors, ↑ increased, ↓ decreased.

One of the most compelling examples of gut-derived molecules linking the gut-liver-heart axis is trimethylamine N-oxide (TMAO) [69]. TMAO is produced from dietary choline and phosphatidylcholine via microbial metabolism to trimethylamine (TMA), which is subsequently oxidized in the liver by flavin-containing monooxygenases (primarily FMO3). Elevated plasma levels of TMAO have been associated with several adverse cardiometabolic outcomes, including subclinical myocardial damage, increased atherosclerotic burden, and elevated cardiovascular mortality [69, 70, 71]. Mechanistically, TMAO impairs reverse cholesterol transport, promotes foam cell formation, enhances platelet hyperreactivity, and induces vascular inflammation [72]. Additionally, TMAO exacerbates insulin resistance, promotes adipose tissue inflammation, and aggravates hepatic steatosis, thereby serving as a molecular bridge between gut dysbiosis, liver dysfunction, and cardiovascular pathology [73, 74]. Altogether, the gut-liver-heart axis underscores a complex and bidirectional interaction between gut microbial ecology and host metabolism, with gut-derived metabolites acting as key messengers in the pathogenesis of obesity-related liver disease and cardiovascular complications (Figs. 1,2). Targeting this axis, through modulation of microbiota composition, dietary interventions, or inhibition of specific microbial metabolic pathways, represents a promising avenue for therapeutic development in cardiometabolic diseases.

### 2.5 Sex Differences and Genetic Predisposition

Sex differences and genetic predisposition play a pivotal role in modulating the onset and progression of MASLD and cardiovascular disease in diabetes [75, 76]. Estrogens exert protective metabolic effects through multiple mechanisms, including improvements in insulin sensitivity, stimulation of glucose uptake in peripheral tissues, and favorable modulation of lipid metabolism with increased high-density lipoprotein (HDL) and reduced LDL cholesterol levels [77]. These actions contribute to the lower prevalence and slower progression of MASLD observed in premenopausal women [78]. Estrogens also attenuate systemic inflammation and oxidative stress, pathways central to both hepatic steatosis and atherosclerotic disease [79]. In contrast, following menopause, the decline in circulating estrogens leads to increased visceral adiposity, impaired lipid handling, and higher inflammation, thereby accelerating the risk of advanced liver disease and cardiovascular complications [80].

In men, androgens may exert divergent effects depending on concentration and context. While physiological androgen levels are associated with improved lean body mass and metabolic efficiency, hypogonadism is frequently observed in men with diabetes and MASLD and is linked to greater visceral adiposity, insulin resistance, and CVD risk [81, 82, 83].

Beyond this hormonal influence, genetic variants such as *PNPLA3* and *TM6SF2*, critically shape disease expression. The *PNPLA3 I148M* polymorphism, strongly associated with hepatic steatosis and fibrosis, also alters systemic lipid partitioning, leading to reduced hepatic very-low-density lipoprotein (VLDL) secretion and increased intrahepatic triglyceride accumulation [84, 85]. Similarly, TM6SF2 E167K variants impair hepatic lipid export, favoring steatosis but paradoxically reducing circulating LDL cholesterol. However, the net effect appears to increase susceptibility to hepatic fibrosis while not fully mitigating cardiovascular risk [84, 85]. Personalized approaches that account for these variables may help refine risk stratification and therapeutic targeting.

### 2.6 The Cardiovascular-Kidney-Metabolic Axis and Its Integration With MASLD

The recent conceptualization of the cardiovascular-kidney-metabolic (CKM) syndrome has reshaped our understanding of systemic metabolic injury, emphasizing that the heart, kidney, and metabolic organs, including the liver, function as an integrated network rather than isolated targets of disease [86]. The kidney plays a pivotal role in this continuum: chronic hyperglycaemia, insulin resistance, oxidative stress, and inflammation drive glomerular hyperfiltration, endothelial dysfunction, and albuminuria, which in turn amplify neurohormonal activation, vascular stiffness, and cardiac remodelling [87, 88]. Parallel mechanisms, such as activation of the renin-angiotensin-aldosterone system and alterations in sodium-glucose transport, further link renal dysfunction to adverse cardiovascular outcomes [89, 90]. Increasing evidence also points to a close bidirectional interaction between CKM dysfunction and MASLD [91]. Renal impairment is frequent in MASLD and independently predicts higher cardiovascular morbidity and mortality, while hepatic inflammation and altered lipid metabolism can accelerate renal microvascular injury [92]. Integrating MASLD within the CKM framework supports a unified view of multi-organ metabolic injury, where the liver acts as both a mediator and a marker of systemic cardiometabolic stress [93]. This perspective advocates for multidimensional screening, including assessment of liver stiffness, kidney function, and subclinical cardiac injury, and for the adoption of therapeutic strategies, such as sodium–glucose cotransporter-2 (SGLT2) inhibitors and GLP-1 receptor agonists, that confer simultaneous cardio-renal-hepatic protection [93, 94].

## 3. Clinical Implications

### 3.1 Common Cardiovascular Manifestations

Cardiovascular disease remains the leading cause of death in individuals with T2D and is increasingly recognized as a major cause of morbidity and mortality among individuals with MASLD [95]. The overlap of these conditions amplifies the risk of coronary artery disease (CAD), heart failure (HF), and arrhythmias, beyond the contribution of classical risk factors such as hypertension, dyslipidaemia, and obesity. Patients often present with atypical or silent symptoms, which complicates timely diagnosis and risk prediction [96, 97]. CAD in diabetes frequently manifests as diffuse and severe coronary atherosclerosis. Due to autonomic neuropathy, typical anginal symptoms may be absent, increasing the likelihood of delayed diagnosis. As a result, diabetic patients carry a two- to fourfold higher risk of cardiovascular death compared with non-diabetic individuals [9, 98]. In MASLD, the prevalence of CAD is lower than in T2D but its severity may be accentuated by cirrhotic cardiomyopathy, where chronic inflammation and altered lipid handling impair cardiac performance [99, 100].

HF is another shared endpoint. In diabetes, persistent hyperglycaemia, oxidative stress, and neurohormonal activation drive myocardial fibrosis and vascular remodelling, leading to increased ventricular stiffness [101, 102]. In liver disease, cirrhotic cardiomyopathy reduces contractile reserve and blunts the haemodynamic response to stress, while a hyperdynamic circulation may conceal early manifestations [103].

Arrhythmias are also frequent. In diabetes, mechanisms include autonomic imbalance, structural remodelling, and electrolyte disturbances, particularly during decompensation or renal dysfunction [104]. In MASLD and cirrhosis, QT interval (QT) prolongation is common and linked to electrolyte shifts and impaired drug metabolism, while advanced disease predisposes to atrial fibrillation through chronic inflammation and high-output states [105].

### 3.2 Diagnostic Challenges

#### 3.2.1 Cardiovascular Risk Scores

MASLD has emerged as an independent cardiovascular risk factor, and its coexistence with T2D defines a particularly high-risk phenotype [106]. For patients with diabetes, tools such as SCORE2-Diabetes provide more refined risk estimation by integrating traditional and diabetes-specific parameters [107]. By contrast, no validated cardiovascular risk score is currently available for MASLD alone [57]. Nonetheless, indices such as fibrosis-4 index (FIB-4) may offer indirect prognostic information, as advanced liver fibrosis correlates with systemic inflammation and a higher incidence of major adverse cardiovascular events (MACE) [57, 108].

#### 3.2.2 Imaging

The increasing recognition of MASLD in diabetes underscores the need for early detection of subclinical cardiovascular dysfunction [109, 110]. Among available tools, speckle-tracking echocardiography and its parameter, global longitudinal strain (GLS), are particularly promising [111, 112]. GLS detects subtle myocardial impairment well before reductions in ejection fraction, and its prognostic value has been confirmed across several populations [113, 114, 115, 116]. This technique could therefore provide an accessible method for stratifying risk in patients with MASLD and/or T2D.

#### 3.2.3 Biomarkers

High-sensitivity cardiac troponin I (hs-cTnI) is a robust biomarker with strong predictive value in diabetes and MASLD [117, 118, 119, 120]. Even within normal ranges, hs-cTnI identifies patients at increased risk of CAD, HF, and mortality, and its levels appear to decline in response to cardioprotective therapies [121]. The use of sex-specific thresholds may further refine prognostication.

#### 3.2.4 Genetic Background

Variants such as *PNPLA3* and *TM6SF2* not only drive MASLD progression but also interact with systemic metabolic and inflammatory pathways [122, 123, 124]. Incorporating genetic risk into screening algorithms may, in the future, allow more personalized cardiovascular risk assessment [125, 126, 127].

#### 3.2.5 Integration of Screening Tools in Clinical Practice

To enhance risk stratification in patients with T2D and MASLD, individual tools should not be considered in isolation but integrated into a structured diagnostic pathway. A practical approach begins with the SCORE2-Diabetes algorithm, which provides a standardized 10-year cardiovascular risk estimation tailored to patients with diabetes (SCORE2-Diabetes Working Group and the European Society of Cardiology [ESC] Cardiovascular Risk Collaboration). SCORE2-Diabetes: 10-year cardiovascular risk estimation in type 2 diabetes in Europe [107]. In parallel, the FIB-4 index can be calculated from routine laboratory data to estimate the likelihood of advanced liver fibrosis and to capture its systemic implications [108]. For patients identified as high risk by either cardiovascular or hepatic assessment, advanced imaging with GLS may detect subclinical myocardial dysfunction before overt heart failure develops [111]. In addition, circulating biomarkers such as hs-cTnI can provide incremental prognostic value, reflecting ongoing myocardial injury even in asymptomatic individuals [120].

By combining these tools into a stepwise algorithm, clinicians can move from broad population-based screening (SCORE2-Diabetes and FIB-4) to targeted advanced testing (GLS and hs-cTnI) in selected high-risk patients. This integrated strategy allows early identification of subclinical organ damage and supports the timely initiation of cardioprotective and hepatoprotective interventions.

### 3.3 Prognostic Implications

The coexistence of MASLD and T2D is associated with a markedly worsened cardiovascular prognosis. Shared mechanisms, including systemic inflammation, insulin resistance, lipotoxicity, and oxidative stress, accelerate atherosclerosis and myocardial dysfunction [39, 120, 128]. Outcomes after acute cardiovascular events are also poorer in this population, with higher rates of hospitalization and mortality [19, 129].

Early identification of high-risk patients is essential. Combining liver fibrosis scores (e.g., FIB-4), imaging modalities (e.g., GLS, coronary calcium scoring), and biomarkers (e.g., hs-cTnI, natriuretic peptides) provides a multidimensional assessment [130]. Such integrated strategies may guide timely interventions, from lifestyle modification to the initiation of cardioprotective drugs [131, 132].

Ultimately, the dual burden of MASLD and T2D requires a multidisciplinary approach. Collaboration among hepatologists, cardiologists, and diabetologists is central to effective management and to reducing the risk of long-term complications [133, 134, 135].

## 4. Management Strategies

### 4.1 Lifestyle Interventions

#### 4.1.1 Diet

Lifestyle modification is the foundation of care in both MASLD and T2D [136, 137]. Among dietary approaches, the Mediterranean diet stands out as the most effective in improving hepatic and cardiometabolic outcomes [138]. Its emphasis on plant-based foods, whole grains, and unsaturated fats supports better glycaemic control, reduced liver fat, and lower cardiovascular risk. Randomized trials confirm that adherence to this dietary pattern not only improves insulin sensitivity but also reduces hepatic steatosis, highlighting its central role in clinical practice [138, 139, 140].

#### 4.1.2 Physical Activity

Regular physical activity is equally important. Aerobic training enhances mitochondrial function and promotes hepatic fat oxidation, while resistance training increases muscle glucose uptake and lean mass [141, 142, 143, 144]. Combining the two modalities maximizes metabolic benefits, with sustained exercise reducing cardiovascular events over time [145].

#### 4.1.3 Weight Reduction

Weight loss is a major therapeutic target. Even modest reductions (5–10%) lead to consistent improvements in steatosis, while greater losses may reverse fibrosis [146, 147, 148]. Structured interventions integrating diet, exercise, and behavioural support are the most effective, though alternative approaches such as intermittent fasting and very-low-calorie diets are gaining interest [149, 150].

### 4.2 Pharmacological Interventions

#### 4.2.1 Diabetes Drugs With Cardiovascular and Hepatic Benefits

4.2.1.1 SGLT2 Inhibitors

SGLT2 inhibitors have demonstrated robust cardiovascular protection in patients with T2D. In the EMPA-REG OUTCOME trial, empagliflozin significantly reduced cardiovascular mortality by 38%, hospitalization for heart failure by 35%, and all-cause mortality by 32% compared with placebo [151]. Similarly, the CANVAS program with canagliflozin reported a 14% relative risk reduction in major adverse cardiovascular events and a 33% reduction in hospitalization for heart failure [152]. These results have also been confirmed in both meta-analyses and real-world studies [153, 154, 155]. Beyond cardiovascular outcomes, the E-LIFT trial demonstrated that empagliflozin reduced liver fat content and improved aminotransferases in patients with T2D and MASLD [156]. Similar hepatic benefits were also observed with dapagliflozin in the EFFECT II randomized trial, which showed a significant reduction in liver fat content assessed by magnetic resonance imaging–proton density fat fraction (MRI-PDFF) and improvement in alanine aminotransferase levels after 12 weeks of treatment [157]. Nevertheless, these findings should be interpreted with caution. The available evidence is largely based on biochemical and imaging endpoints, while robust histological confirmation of fibrosis improvement with SGLT2 inhibitors is still lacking.

4.2.1.2 GLP-1 Receptor Agonists

GLP-1 receptor agonists have also proven effective in reducing cardiovascular risk [158]. The LEADER trial showed that liraglutide reduced the risk of major adverse cardiovascular events by 13% and cardiovascular death by 22% [159]. In SUSTAIN-6, semaglutide reduced MACE by 26% [160], while the REWIND trial with dulaglutide demonstrated a 12% reduction, even in a population with predominantly primary prevention [161]. In addition, the phase 2 LEAN trial showed that liraglutide promoted histological resolution of metabolic dysfunction-associated steatohepatitis (MASH) in patients with biopsy-proven disease [162]. While imaging studies and liver enzyme improvements are encouraging, histological evidence remains limited. The LEAN trial provided proof-of-concept for liraglutide [162], but large-scale outcome studies are needed before firm conclusions on fibrosis regression can be drawn. Consistent with these findings, exploratory analyses from the AWARD and REWIND programs showed that dulaglutide reduced liver fat content and aminotransferase levels, while confirming significant reductions in major adverse cardiovascular events in patients with type 2 diabetes [163]. Furthermore, in a phase 2 randomized trial, semaglutide demonstrated significant histological resolution of MASH without worsening of fibrosis, along with reductions in liver fat and aminotransferases [164].

In a global TriNetX analysis of nearly 19,000 semaglutide-treated MASLD patients reported improved one-year survival, lower cardiovascular risk, and reduced progression to advanced liver disease compared with matched controls, with benefits attributed to improvements in body mass index (BMI), lipid profile, glycated hemoglobin (HbA1c), and systemic inflammation [165]. A second TriNetX comparative study involving more than 640,000 patients with T2D and non-alcoholic fatty liver disease (NAFLD) showed that semaglutide was associated with a significantly lower risk of major adverse liver outcomes, including decompensated cirrhosis, hepatocellular carcinoma, and liver transplantation, compared with SGLT2 inhibitors, dipeptidyl peptidase-4 (DPP-4) inhibitors, and thiazolidinediones, and also conferred a survival advantage [166].

4.2.1.3 Tirzepatide

Tirzepatide, a dual glucose-dependent insulinotropic polypeptide (GIP) and GLP-1 receptor agonist, has shown remarkable efficacy in the SURPASS program. In SURPASS-2, tirzepatide achieved HbA1c reductions of up to –2.3% and weight loss exceeding 11 kg compared with semaglutide [167]. Pooled analyses suggest additional cardiovascular benefits through improvements in blood pressure, triglycerides, and inflammatory markers [168, 169]. The ongoing SURPASS-CVOT will provide definitive evidence regarding cardiovascular outcomes, and preliminary not peer reviewed results showed non-inferiority compared to dulaglutide [170]. Furthermore, in the SURMOUNT-1 trial, tirzepatide led to substantial weight loss (up to –21% of baseline body weight) and improvements in hepatic steatosis assessed by MRI-PDFF [171]. Although tirzepatide has shown striking metabolic and imaging-based hepatic benefits, histological data are still preliminary and experimental [172]. Results from phase 2 studies such as SYNERGY-NASH are promising but require confirmation in larger and longer-term trials [173, 174].

#### 4.2.2 Lipid-Lowering Therapies

Statins remain first-line therapy for dyslipidaemia in patients with T2D and MASLD. Despite past concerns about hepatotoxicity, they are safe and reduce cardiovascular morbidity and mortality, with possible antifibrotic effects [175, 176]. Proprotein convertase subtilisin/kexin type 9 (PCSK9) inhibitors represent an additional option for high-risk patients, with strong low-density lipoprotein cholesterol (LDL-C) lowering efficacy and growing evidence of potential benefits on hepatic lipid injury [177, 178].

### 4.3 Anti-Inflammatory and Antifibrotic Agents

Novel therapies are under investigation to target the overlapping inflammatory and fibrotic pathways of MASLD and CVD. Obeticholic acid, a FXR agonist, has improved histology in MASH and could help slow fibrosis [179]. Other anti-inflammatory and antifibrotic drugs are being developed and may become valuable for managing this dual burden [180].

### 4.4 Bariatric Surgery

Bariatric surgery is the most effective intervention for patients with severe obesity and metabolic complications. Procedures such as Roux-en-Y gastric bypass and sleeve gastrectomy produce long-term weight loss and robust improvements in insulin sensitivity [181, 182]. They can reverse hepatic steatosis and, in some cases, fibrosis, with additional benefits on cardiovascular outcomes [183, 184]. Beyond weight reduction, changes in gut hormones, bile acid signalling, and microbiota contribute to these effects. Surgery should be considered for selected patients with advanced disease who fail lifestyle and pharmacological therapy [148]. When considering metabolic/bariatric surgery in patients with T2D and MASLD, careful patient selection is essential. Current guidelines recommend surgery for individuals with a BMI ≥40 kg/m^2^, or ≥35 kg/m^2^ in the presence of obesity-related comorbidities such as uncontrolled T2D, advanced MASLD, or cardiovascular disease [185]. Optimal candidates are those who fail to achieve durable weight loss or metabolic control with lifestyle and pharmacological therapies, and who can adhere to long-term nutritional and medical follow-up. However, surgery is not without risk. Early postoperative complications include bleeding, anastomotic leak, and venous thromboembolism, while long-term issues may involve micronutrient deficiencies, hypoglycemia, dumping syndrome, and surgical revisions [186]. In addition, psychosocial assessment is recommended to evaluate readiness for surgery and ensure multidisciplinary support [187]. These considerations highlight the importance of balancing expected cardiometabolic and hepatic benefits against procedural risks, tailoring the approach to individual patient profiles.

All current and emerging interventions targeting MASLD and type 2 diabetes are summarized in Table 1 (Ref. [138, 139, 140, 141, 142, 143, 144, 145, 146, 147, 148, 149, 150, 151, 152, 153, 154, 155, 156, 157, 158, 159, 160, 161, 162, 163, 164, 165, 166, 167, 168, 169, 170, 171, 172, 173, 174, 175, 176, 177, 178, 179, 180, 181, 182, 183, 184, 185, 186, 187]), while the most relevant clinical trials investigating SGLT2 inhibitors, GLP-1 receptor agonists, and tirzepatide in MASLD and CVD are summarized in Table 2 (Ref. [156, 157, 162, 163, 164, 167, 168, 169, 170]).

**Table 1.** **Current and emerging strategies in the management of MASLD and type 2 diabetes**.

Category	Intervention	Primary outcome type	Key outcomes	Additional cardiometabolic outcomes	Ref.
Lifestyle interventions	Mediterranean Diet	Clinical/biochemical	↓ Liver fat, ↑ insulin sensitivity, improved glycaemic control	↓ CV risk factors, ↓ inflammation	[138, 139, 140]
	Physical Activity (aerobic, resistance, combined)	Clinical/biochemical, CV outcomes	↓ Insulin resistance, ↓ hepatic steatosis	Improved cardiorespiratory fitness, ↓ CV events with sustained activity	[141, 142, 143, 144, 145]
	Weight Loss (5–10%)	Histological, clinical	Significant ↓ steatosis; >10% may reverse fibrosis	↓ HbA1c, improved lipid profile	[146, 147, 148]
	Intermittent Fasting/VLCD	Clinical/biochemical	Accelerated ↓ liver fat, improved glycaemic control	Potential improvements in BP and systemic inflammation	[149, 150]
Surgical interventions	Bariatric Surgery	Histological, clinical, CV outcomes	Sustained weight loss, improved insulin sensitivity, ↓ liver fat and fibrosis	↓ HbA1c, ↓ CV events, possible fibrosis reversal in MASH	[181, 182, 183, 184, 185, 186, 187]
Pharmacological interventions	SGLT2 Inhibitors	Imaging, biochemical, CV outcomes	↓ HbA1c, ↓ hepatic steatosis	↓ MACE, ↓ HF hospitalization, ↓ CV mortality	[151, 152, 153, 154, 155, 156]
	GLP-1 Receptor Agonists	Imaging, histological, CV outcomes	↓ HbA1c, weight loss, ↓ liver fat and fibrosis markers	↓ MACE in high-risk patients	[157, 158, 159, 160, 161, 162, 163, 164, 165, 166]
	Tirzepatide (GIP/GLP-1 RA)	Imaging, biochemical	Superior ↓ HbA1c, weight, ↓ liver fat	Potential histological MASH improvement	[167, 168, 169, 170, 171, 172, 173, 174]
	Statins	Biochemical, CV outcomes	Safe in MASLD, ↓ LDL, possible antifibrotic effect	↓ CV events, ↓ mortality	[175, 176]
	PCSK9 Inhibitors	Biochemical, CV outcomes	↓ LDL, ↓ systemic inflammation	Possible benefit in lipid-driven hepatic injury, ↓ ASCVD risk	[177, 178]
	Anti-inflammatory/Antifibrotic Agents (e.g., obeticholic acid)	Histological	↓ Fibrosis progression in MASH	Potential CV benefit under investigation	[179, 180]

ASCVD, atherosclerotic cardiovascular disease; BP, blood pressure; CV, cardiovascular; GIP, glucose-dependent insulinotropic polypeptide; GLP-1 RA, glucagon-like peptide-1 receptor agonist; HbA1c, glycated hemoglobin; HF, heart failure; LDL, low-density lipoprotein; MACE, major adverse cardiovascular events; MASLD, metabolic dysfunction-associated steatotic liver disease; MASH, metabolic dysfunction-associated steatohepatitis; PCSK9, proprotein convertase subtilisin/kexin type 9; RA, receptor agonist; SGLT2, sodium–glucose cotransporter-2; VLCD, very-low-calorie diet; ↑ increased; ↓ decreased.

**Table 2.** **Key clinical trials of SGLT2i, GLP-1 RAs, and tirzepatide in MASLD and cardiovascular disease**.

Drug/Class	Representative trial(s)	Primary MASLD/MASH outcome	Liver enzymes	CV outcomes	Ref.
Empagliflozin (SGLT2i)	E-LIFT	↓ hepatic fat fraction	↓ ALT/AST	↓ CV risk surrogates; large CVOT shows ↓ MACE	[156]
Dapagliflozin (SGLT2i)	EFFECT II	↓ liver fat (MRI-PDFF)	↓ ALT	Benefits on CV/renal outcomes in class	[157]
Liraglutide (GLP-1 RA)	LEAN	↑ MASH resolution, ↓ progression of fibrosis	↓ ALT	CV benefit in class	[162]
Semaglutide (GLP-1 RA)	Phase 2 NASH study	↑ MASH resolution without worsening fibrosis	↓ ALT/AST	↓ MACE in T2DM CVOT	[164]
Dulaglutide (GLP-1 RA)	AWARD program	↓ liver fat (exploratory)	↓ ALT	↓ CV events in CVOT (class)	[163]
Tirzepatide (GIP/GLP-1 RA)	SURPASS metabolic trials	↓ liver fat (MRI-PDFF)	↓ ALT/AST	↓ weight/BP/lipids; CVOT ongoing, preliminary results show non-inferiority to dulaglutide	[167, 168, 169, 170]

ALT, alanine aminotransferase; AST, aspartate aminotransferase; BP, blood pressure; CV, cardiovascular; CVOT, cardiovascular outcomes trial; GIP, glucose-dependent insulinotropic polypeptide; GLP-1 RA, glucagon-like peptide-1 receptor agonist; MASLD, metabolic dysfunction-associated steatotic liver disease; MACE, major adverse cardiovascular events; MRI-PDFF, magnetic resonance imaging–proton density fat fraction; MASH, metabolic dysfunction-associated steatohepatitis; SGLT2i, sodium–glucose cotransporter-2 inhibitor; T2DM, type 2 diabetes mellitus; ↑ increased; ↓ decreased.

### 4.5 Incorporating Telemedicine and Digital Health Tools

The rapid evolution of digital health technologies has significantly transformed the landscape of chronic disease management, including metabolic disorders such as type 2 diabetes and MASLD [188]. Telemedicine platforms facilitate remote consultations, enabling timely medical interventions and continuity of care, particularly for patients with limited access to healthcare facilities [189]. Digital tools such as continuous glucose monitoring systems and mobile health applications support real-time tracking of glycaemic control, dietary intake, and physical activity. Wearable devices further empower patients by providing actionable feedback and fostering greater adherence to lifestyle interventions [190, 191]. Moreover, the integration of artificial intelligence into digital health systems enhances personalized care through predictive analytics, enabling more accurate risk stratification and optimization of treatment strategies [192].

## 5. Preventive Strategies

### 5.1 Primary Prevention

In individuals at high metabolic risk, early prevention is crucial to halt the progression toward T2D, MASLD, and CVD. Lifestyle changes remain the most effective first-line strategy. Smoking cessation markedly lowers cardiovascular risk and reduces all-cause mortality [193]. Limiting alcohol intake, increasing physical activity, and adopting a balanced dietary pattern such as the Mediterranean diet are also associated with improvements in glycaemic control, lipid profile, and systemic inflammation [194, 195, 196, 197].

Primary prevention aims to intervene before structural damage occurs [198]. Intensive dietary counseling shortly after a T2D diagnosis improves metabolic outcomes, while even modest weight reduction (≈5%) translates into lower blood pressure, improved lipid values, and better glucose control [199]. No single dietary approach is universally optimal, but low-carbohydrate and Mediterranean-style diets are among the most effective in reducing HbA1c and body weight [200, 201]. Structured exercise programs complement these effects, lowering HbA1c by about 0.6%, improving cardiovascular fitness, and enhancing psychological well-being [202, 203].

Lifestyle modification should therefore represent the foundation of early care [204]. Nevertheless, in patients with multiple risk factors or established end-organ involvement, pharmacological treatment needs to be introduced promptly to achieve comprehensive risk reduction [198].

#### Screening for MASLD and CVD

Given the strong interconnection between MASLD, T2D, and cardiovascular complications, systematic screening is essential. Patients with T2D should be regarded as at least at equivalent risk to those with established coronary disease. The SCORE2-Diabetes algorithm refines 10-year risk prediction by integrating traditional and diabetes-specific factors, including HbA1c and renal function [107].

In practice, CAD may be suspected even in the absence of chest pain, which is frequently absent in diabetes [98]. Dyspnoea on exertion is an important warning sign [205], particularly in the presence of multiple risk factors or abnormal electrocardiogram (ECG) findings. Stress testing in this setting can help identify patients who might benefit from revascularization [199]. For MASLD, ultrasound remains the first-line diagnostic tool owing to its accessibility and cost-effectiveness. Transient elastography (FibroScan®), particularly with the Controlled Attenuation Parameter (CAP), provides additional non-invasive information on liver stiffness and fat content [206]. Several biochemical scores, such as the Fatty Liver Index, Hepatic Steatosis Index, and NAFLD Liver Fat Score, are also useful for risk stratification and can be integrated into routine practice [207, 208].

Studies have demonstrated that participation in comprehensive cardiac rehabilitation is associated with lower rates of rehospitalization, improved cardiovascular risk factor control, and enhanced quality of life, underscoring the vital role of these programs in the continuum of care for individuals with cardiovascular disease (Table 3, Ref. [193, 194, 195, 196, 197, 198, 199, 200, 201, 202, 203, 209, 210, 211, 212, 213, 214, 215, 216, 217, 218, 219, 220, 221, 222, 223, 224]) [225, 226, 227].

**Table 3.** **Integrated strategies for primary, secondary, and tertiary prevention of cardiovascular disease in patients with T2D and MASLD**.

Category	Key interventions and concepts	Drug classes	References
Primary prevention	Risk Factor Modification		
Diet	- Intensive dietary intervention at diabetes diagnosis improves glycemic control.	-	[194, 195, 196, 197, 198, 199, 200, 201]
	- A low-carbohydrate, Mediterranean-style diet yields the greatest short-term reductions in HbA1c and body weight.		
Physical activity	- Improves cardiovascular risk factors, enhances well-being, supports weight loss, ↓ HbA1c (~0.6%). Recommended: ≥150 min/week of moderate-intensity aerobic activity plus resistance training 2–3 times per week.	-	[202, 203]
Smoking cessation	- Smoking is strongly associated with increased cardiovascular events and all-cause mortality.	-	[193]
In high risk individuals	- Multifactorial pharmacological intervention to manage all risk factors not at target.	Individualized	[199]
Secondary/Tertiary prevention	Comprehensive Management in Established Disease		
Lipid management	- LDL-C targets <70 mg/dL for high-risk patients.	First-line: high-intensity statins. Add-on: ezetimibe, PCSK9 inhibitors, bempedoic acid, or inclisiran as needed.	[209]
	- LDL-C targets <55 mg/dL for very-high-risk individuals.		
Blood pressure control	- <140/90 mmHg in most patients.	Preferred: ACEi/ARB, especially with proteinuria.	[209]
	- <130/80 mmHg in individuals with diabetes or very high cardiovascular risk.		
Glycemic control	- For most patients, an HbA1c <7% is recommended.	Metformin (first-line), add SGLT2i, GLP-1 RA.	[209, 210, 211]
		Prioritize SGLT2i and/or GLP-1 RA in patients with CVD, HF, CKD regardless of baseline HbA1c.	
Renal protection	- Regular monitoring of kidney function (eGFR and albuminuria) is critical.	Use ACEi/ARB; consider SGLT2i and finerenone in diabetic kidney disease.	[209, 211, 212, 213]
Antiplatelet therapy	- Low-dose aspirin for secondary prevention.	Aspirin ± P2Y12 inhibitor.	[214, 215]
	- Dual therapy post-ACS/PCI.		
Cardiac rehabilitation and lifestyle clinics	- Initiated during hospitalization.	-	[216, 217, 218, 219, 220, 221, 222, 223, 224]
	- Multidisciplinary follow-up optimizes therapy and risk factor control.		

ACEi, angiotensin-converting enzyme inhibitor; ACS acute coronary syndrome; ARB, angiotensin II receptor blocker; CKD, chronic kidney disease; CV, cardiovascular; CVD, cardiovascular disease; eGFR, estimated glomerular filtration rate; GLP-1 RA, glucagon-like peptide-1 receptor agonist; HbA1c, glycated hemoglobin; HF, heart failure; LDL-C, low-density lipoprotein cholesterol; MASLD, metabolic dysfunction-associated steatotic liver disease; PCI, percutaneous coronary intervention; PCSK9, proprotein convertase subtilisin/kexin type 9; SGLT2i, sodium–glucose cotransporter-2 inhibitor; T2D, type 2 diabetes.

### 5.2 Secondary and Tertiary Prevention

#### 5.2.1 Comprehensive Care in Established Disease

For patients with diagnosed CVD, secondary and tertiary prevention strategies are essential to reduce recurrence and improve survival. This requires an integrated approach addressing all modifiable risk factors [225, 228].

- Lipid management. LDL-C lowering is central to prevention, with stringent targets (<70 mg/dL in high-risk, <55 mg/dL in very-high-risk individuals) [209]. Statins remain first-line therapy; ezetimibe, PCSK9 inhibitors, and bempedoic acid can be added if targets are not achieved [209, 228, 229, 230].
- Blood pressure. In diabetes, levels <130/80 mmHg are recommended when tolerated. Angiotensin-converting enzyme (ACE) inhibitors or angiotensin II receptor blockers (ARBs) are preferred, particularly in proteinuric kidney disease [209].
- Glycaemic control. Most patients should aim for HbA1c <7%. For those with established CVD, agents with proven cardiovascular benefit, notably SGLT2 inhibitors and GLP-1 RAs, should be prioritized regardless of baseline glycaemia [209, 210, 211].
- Renal protection. Monitoring estimated glomerular filtration rate (eGFR) and albuminuria is mandatory. In addition to renin-angiotensin system (RAS) blockade, SGLT2 inhibitors and finerenone offer significant cardio-renal benefits [212, 213].
- Antiplatelet therapy. Low-dose aspirin remains standard for secondary prevention unless contraindicated. Dual antiplatelet therapy may be indicated after acute coronary syndrome or percutaneous coronary intervention (PCI), with duration tailored to ischemic and bleeding risks [214, 215].

#### 5.2.2 Cardiac Rehabilitation and Lifestyle Clinics

Following a cardiovascular event, cardiac rehabilitation is a cornerstone of long-term care. Early mobilization during hospitalization shortens recovery and improves functional capacity. Subsequent structured programs combine aerobic and resistance training with education on medication adherence, nutrition, smoking cessation, and stress management [216, 217, 218, 219, 220, 221]. The benefits extend beyond physical recovery. Addressing psychological aspects, such as depression and anxiety, is critical to ensure adherence and quality of life [221]. Multidisciplinary follow-up with cardiologists, nurses, physiotherapists, dietitians, and psychologists helps maintain progress and prevent relapses. Telemedicine and digital health platforms can expand access, particularly in rural or resource-limited settings [222]. Participation in comprehensive rehabilitation has been consistently associated with reduced rehospitalizations, better risk factor control, and improved survival, underscoring its importance as part of secondary and tertiary prevention (Table 3) [223, 224].

## 6. Research Gaps and Future Directions

### 6.1 Unanswered Questions

Although the combined burden of T2D, MASLD, and CVD is increasingly recognized, several aspects remain poorly defined. The mechanistic pathways that link hepatic inflammation, metabolic dysregulation, and cardiovascular remodelling are still only partially understood [231, 232]. This is particularly evident in lean or normal-weight individuals with T2D and MASLD, in whom conventional risk factors do not fully explain disease progression [233, 234]. More translational studies are needed to clarify these interactions and to identify early drivers of risk.

### 6.2 Emerging Trends

The search for reliable biomarkers and non-invasive tools represents a priority. While genetic variants, circulating markers, and advanced imaging techniques have shown potential, their integration into routine practice requires broader validation across different populations and healthcare settings [235]. Precision medicine approaches, supported by large-scale “omics” studies, may allow a more individualized assessment of risk. At the same time, artificial intelligence and machine learning could facilitate the analysis of complex datasets that combine genetic, imaging, and clinical information [236]. However, these technologies must be rigorously tested in real-world scenarios before being adopted in clinical workflows.

### 6.3 Call for Collaboration

Progress in this field is hindered by fragmented research. Large, multicenter, and interdisciplinary collaborations are needed to ensure reproducibility and generalizability [237]. The active involvement of hepatologists, endocrinologists, cardiologists, and data scientists will be essential to identify prognostic markers, refine integrated risk scores, and test innovative therapeutic strategies. Only through such coordinated efforts can we develop patient-centered models of care that address the full spectrum of cardiometabolic disease.

## 7. Conclusions

The rising prevalence of T2D, MASLD, and CVD is not only a public health issue but also a daily clinical reality. Patients increasingly embody this “triad,” often presenting with overlapping risk factors and accelerated disease trajectories. While substantial progress has been made in understanding pathophysiology and testing new therapies, current practice still suffers from fragmented care.

Our perspective is that cardiologists, hepatologists, and diabetologists must move beyond traditional silos. A shared care model, built on integrated risk stratification and combined therapeutic strategies, is essential. At the same time, unanswered questions, ranging from the long-term impact of dual incretin therapies to the clinical implementation of AI-based prediction models, demand collaborative, multicenter research. Ultimately, a shift in mindset is required: these patients should not be viewed as having three separate diseases but rather as carrying a unified cardiometabolic risk that calls for unified solutions.
